# Supplementary figures and images for: Complete chloroplast genome sequence of Adenophora racemosa (Campanulaceae): Comparative analysis with congeneric species
Source: PLoS One. 2021 Mar 18;16(3):e0248788. doi: 10.1371/journal.pone.0248788 (PMC7971521; doi:10.1371/journal.pone.0248788)

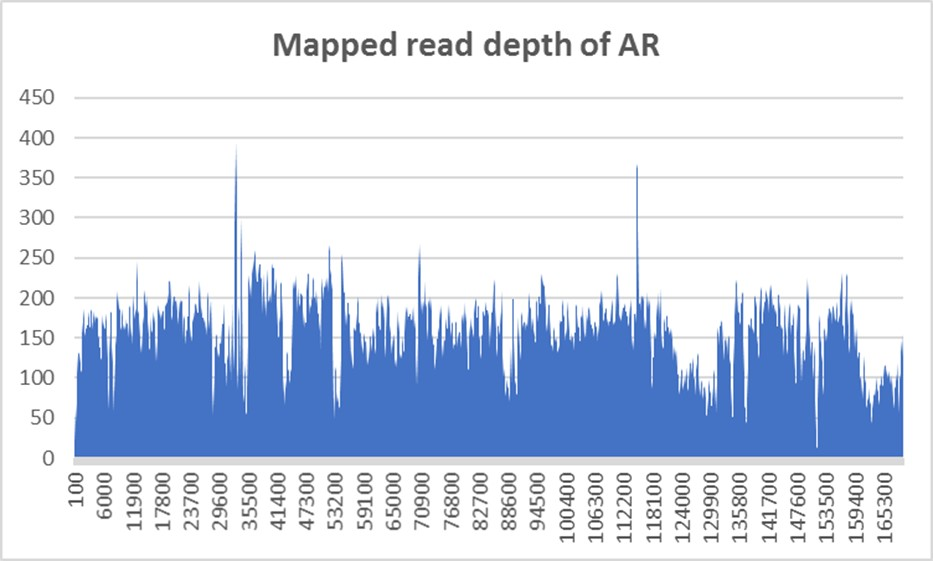

Supplement: S1 Fig — (TIFF) [file pone.0248788.s001.tiff]
