# Supplementary material for: Complete chloroplast genome sequence of Adenophora racemosa (Campanulaceae): Comparative analysis with congeneric species
Source: PLoS One. 2021 Mar 18;16(3):e0248788. doi: 10.1371/journal.pone.0248788 (PMC7971521; doi:10.1371/journal.pone.0248788)
Supplement: S1 Table — (DOCX) [file pone.0248788.s002.docx]

**Table S2. The GenBank accession numbers of all the 13 chloroplast genomes used for phylogenetic analysis.**

| Taxon | GenBank Accession No. |
| --- | --- |
| *Adenophora erecta* | KX462130 |
| *Adenophora divaricata* | KX462129 |
| *Adenophora racemosa* | MT012303 |
| *Adenophora remotiflora* | KP889213 |
| *Adenophora stricta* | KX462131 |
| *Campanula punctata* | KU198434 |
| *Campanula takesimana* | KP006497 |
| *Codonopsis minima* | KY587457 |
| *Platycodon grandiflorum* | KX352464 |
| *Trachelium caeruleum* | EU090187 |
| *Lobelia chinensis* | MF061186 |
